# Correction: Polariton chemistry: controlling molecular dynamics with optical cavities

**DOI:** 10.1039/d0sc90242j

**Published:** 2020-11-09

**Authors:** Raphael F. Ribeiro, Luis A. Martínez-Martínez, Matthew Du, Jorge Campos-Gonzalez-Angulo, Joel Yuen-Zhou

**Affiliations:** Department of Chemistry and Biochemistry, University of California San Diego La Jolla California 92093 USA joelyuen@ucsd.edu

## Abstract

Correction for ‘Polariton chemistry: controlling molecular dynamics with optical cavities’ by Raphael F. Ribeiro *et al.*, *Chem. Sci.*, 2018, **9**, 6325–6339, DOI: 10.1039/C8SC01043A.

The authors regret that incorrect values are reported in Table 1 of the original article. The corrected Table 1 is shown below.

**Table 1:** Timescales relevant for the description of organic (*J*-aggregate) microcavity relaxation dynamics at room temperature^a^

Process	Initial state(s)	Final state(s)	Timescale	Ref. in original article	Ref. in this Correction
Rabi oscillations	—	—	15–80 fs (50–300 meV)	93	1
Cavity leakage	Cavity photon	—	35–100 fs	94–96	2–4
Vibrational relaxation	UP	Dark states	∼50 fs	11	5
	Dark states	LP	∼10 ps	99	6
Photoluminescence	UP	—	∼100 fs	95	3
	LP	—	∼100 fs	95	3
	Bare exciton	—	∼1–100 ps	94, 100 and 101	2, 7 and 8

a In typical organic dyes, vibrational relaxation following electronic excitation occurs on the order of 10–1000 fs.^9,10^

The Royal Society of Chemistry apologises for these errors and any consequent inconvenience to authors and readers.
